# Features of Effective T Cell-Inducing Vaccines against Chronic Viral Infections

**DOI:** 10.3389/fimmu.2018.00276

**Published:** 2018-02-16

**Authors:** Eleni Panagioti, Paul Klenerman, Lian N. Lee, Sjoerd H. van der Burg, Ramon Arens

**Affiliations:** ^1^Department of Medical Oncology, Leiden University Medical Center, Leiden, Netherlands; ^2^Department of Immunohematology and Blood Transfusion, Leiden University Medical Center, Leiden, Netherlands; ^3^Nuffield Department of Medicine, University of Oxford, Oxford, United Kingdom

**Keywords:** T cells, quality, vaccine, prophylaxis, chronic infection, virus

## Abstract

For many years, the focus of prophylactic vaccines was to elicit neutralizing antibodies, but it has become increasingly evident that T cell-mediated immunity plays a central role in controlling persistent viral infections such as with human immunodeficiency virus, cytomegalovirus, and hepatitis C virus. Currently, various promising prophylactic vaccines, capable of inducing substantial vaccine-specific T cell responses, are investigated in preclinical and clinical studies. There is compelling evidence that protection by T cells is related to the magnitude and breadth of the T cell response, the type and homing properties of the memory T cell subsets, and their cytokine polyfunctionality and metabolic fitness. In this review, we evaluated these key factors that determine the qualitative and quantitative properties of CD4^+^ and CD8^+^ T cell responses in the context of chronic viral disease and prophylactic vaccine development. Elucidation of the mechanisms underlying T cell-mediated protection against chronic viral pathogens will facilitate the development of more potent, durable and safe prophylactic T cell-based vaccines.

## Introduction

Our bodies are persistently exposed to various pathogens present in the environment. The immune system is fortified with physical barriers and with diverse immune cell populations that play an integral role in protection against disease. Long-term immune responses are mediated by antigen-specific lymphocytes and antibodies that are formed upon pathogen entry. Memory B and T cells are numerically and functionally superior to their naïve precursors cells that are present before infection, and upon encounter with the same pathogen memory immune cells are able to induce a more rapid and powerful recall response (i.e., immunological memory) (1, 2).

The majority of prophylactic vaccines against viral infections have focused on the induction of neutralizing antibodies. Indeed, potent antibody inducing vaccines against virally induced diseases are available. Nevertheless, they fail to provide long-term efficacy and protection against a number of chronic viral infections. Studies in mice, non-human primates, and humans provide evidence that effective prophylactic vaccines against chronic (low level and high level) replicating viruses [i.e., herpesviruses, human immunodeficiency virus (HIV), and hepatitis C virus (HCV)] should engage strong cellular T cell immunity (3–5). The development of T cell-eliciting prophylactic vaccines has gained increasing attention, although such vaccines are not always able to provide sterilizing immunity. Despite various promising vaccines that are capable of stimulating robust T cell responses, the critical factors of T cell-mediated immune protection against these chronic infections have not been clearly defined. Often, the memory response provoked by vaccines is not sustained and frequently diminishes over time (6, 7). Thus, more knowledge is required to tailor the vaccine’s capacity to induce durable CD4^+^ and/or CD8^+^ T cell responses of appropriate magnitude and quality to effectively contribute to pathogen clearance. Elucidating the mechanisms through which antigen-specific T cell populations mediate long-term protection against viruses at body surfaces and (lymphoid) tissues remains an important goal, and will facilitate the development of more effective and safe prophylactic T cell-eliciting vaccines. Here, we review determinants and mechanistic factors of effective T cell populations implicated in the vaccine efficacy against chronic viral infections, and discuss how this knowledge can be utilized to maximize the possibility of creating effective vaccine platforms for persistent viral infections.

## The Complexity of the Antigen-Specific T Cell Response During Infection

The antigen-specific interactions between T cells and DCs resulting in activation may initially be short lived, before stabilizing and may last up to 12 h. During this period, T cells receive their necessary activating signals (8, 9). For proper activation of naïve CD4^+^ and CD8^+^ T cells, cognate antigenic signals through the TCR (signal 1), costimulatory signals (signal 2) and signals provided by inflammatory cytokines (signal 3) are required (10, 11). Expression of particular chemokine receptors such as CCL19 and CCL21 enhance immune responses by stimulating the interactions between T cells and DCs during antigen presentation (12–15). In addition, the secretion of chemokines by activated DCs and CD4^+^ T cells enhances CD8^+^ T cell accumulation and help attract rare antigen-specific T cells (16, 17). The activation of T cells results in alteration of the expression of various molecules including integrins, selectins, and chemokine receptors, leading to the modulation of key intracellular signaling events that promote proliferation, differentiation, and migration of T cells to inflamed tissues (18–20).

After resolution of the infection, the majority (90–95%) of the effector T cells are eliminated due to programmed cell death and only a small, yet diverse pool of memory cells remains (21, 22). Traditionally, memory T cells were classified into two major categories based on their proliferation capacity, phenotypic features, and migration potential (23). Effector-memory T (T_EM_) cells are identified based on combined expression and/or lack of certain cell surface markers including KLRG1^hi^/CD44^hi^/CD127^lo^/CD62L^lo^. These cells have limited proliferation capacity upon TCR triggering but rapidly produce effector molecules and cytokines such as IFN-γ and TNF (24, 25). Central-memory T (T_CM_) cells are distinguished by the expression of KLRG1^lo^/CD44^hi^/CD127^hi^/CD62L^hi^ surface markers, exhibit a superior proliferation capacity and produce cytokines that are directly associated with better secondary expansion such as interleukin (IL)-2. Secondary lymphoid organs are the main homing sites of T_CM_ cells whereas T_EM_ cells are more dominantly present in (non-lymphoid) tissues (26–29). Both T_CM_ and T_EM_ cells can circulate, whereas a recently discovered new category of T cells present in tissues lacks migratory capacity. These cells, named tissue-resident memory T (T_RM_) cells, permanently reside in peripheral tissues, even after the infection is cleared. T_RM_ cells are present in most organs and tissues and can be defined based on the expression of CD69^hi^/CD62L^lo^/CD44^hi^ and other surface markers (e.g., CD11a, CD38, CD49a, CD103, and CXCR3) (30–33). However, the composition of these markers depends on tissue-specific cues, and expression levels vary in different tissues. Besides these three main memory T cell subsets, a small subset of memory T cells exists that exhibit advanced stem cell like qualities and proliferation capacities compared with other T cell subsets (34). These memory T cells, which were designated stem cell memory T cells (T_SCM_ cells), display a phenotype highly similar to naïve T cells (T_N_ cells), being KLRG1^lo^/CD44^lo^/CD127^hi^/CD62L^hi^/CD69^lo^, but also co-express stem cell antigen (Sca-1), the β chain of the IL-2 and IL-15 receptor (CD122 and IL-2Rβ), and the chemokine receptor CXCR3 (34–39). Some studies reported that T cells with an early stage of differentiation can be induced by vaccines (40, 41) but whether this induction is important for vaccine efficacy is unclear. Thus, whether sufficient amounts of T_SCM_-like T cells able to elicit protection can be generated by vaccines needs further exploration. Notably, humans and mice have broadly analogous T cell biology, and the above described subsets (i.e., T_CM_, T_EM_, T_RM_, and T_SCM_ cells) have been described in both species and share similar characteristics.

Live attenuated as well as synthetic or subunit vaccines are able to elicit T_CM_, T_EM_, and T_RM_ cells (30, 32). With respect to live attenuated vaccines, the vaccine-induced T cell subsets can be highly similar to those subsets that develop upon infection (42). However, live vaccines have disadvantages (e.g., transformation to a virulent form and requires refrigeration), which prompts the development of inactivated, synthetic, or subunit vaccines. T cell subsets that develop upon immunization with those vaccines are highly dependent on the addition of adjuvants and on the route of administration (43).

## The Magnitude of the T Cell Response is Important for Optimal Protection

The magnitude of viral-specific T cell responses is highly dictated by the infectious dose and route of infection (44). Higher infectious dosages lead generally to higher peak values of effector T cells, and correspondingly larger amounts of memory T cells in the circulation are found. However, if the immune system is overwhelmed and virus replication remains at a high level, this eventually leads to exhaustion of T cells and poor memory formation (45).

Given the frequently observed correlation between the magnitude of T cell responses and establishment of immunity during infections, simply determining the magnitude of the vaccine-elicited T cell response may already serve as a predictor of efficacy in vaccination settings. A number of studies have shown a direct association between the vaccine-elicited T cell response size and the ability for virus control (5, 46–48). Several parameters directly impact the magnitude of the vaccine-induced T cell response. In the case of live (attenuated) viruses, the size of the initial dose of the inoculum correlates to the magnitude of the vaccine-specific T cell response until a threshold is reached (49). To reach similar levels as that elicited by virulent virus, inoculum sizes are generally higher for replication-deficient or single-cycle viral vectors. For synthetic vaccines, however, the saturation threshold may not be reached because of lack of sufficient inflammatory signals. However, recent discoveries in adjuvant development and synthetic (nano)particles provide promising approaches to augment T cell responses (50–52). Besides the initial inoculum dosage, booster vaccine regimens increase the magnitude of the T cell response (43, 53, 54) and are likely essential for the majority of vaccine platforms including live vaccines (55). In this regard, vaccines that prime with DNA or adenoviral vectors and boost with modified vaccinia Ankara are excellent demonstrations that underline the supremacy of prime-boost vaccination regimens (4, 56–64).

## Memory T Cell Inflation Provoked by Recombinant Vaccines

An alternative mechanism leading to a durable increased magnitude of memory T cells, described as memory “inflation” (65, 66), is observed for certain viral-specific responses following infection by cytomegalovirus (CMV). Here, antigen-specific T cells specific to a subset of viral peptides show an unusual response, whereby they expand gradually over time and are maintained at high frequencies as T_EM_-like populations—as opposed to the standard expansion and contraction kinetic of conventional memory cells. Critically, and unlike exhausted CD8 T cells that develop during other persistent infections these inflationary responses maintain their effector functions, tissue homing ability and can provide protection against pathogen rechallenge. Memory inflation has also been observed for CMV-specific antibodies, whose levels gradually increase over time (67). Although the rules that determine the onset of memory inflation have not been fully defined, it is clear that for inflation to occur viral antigen must persist long term, a criterion fulfilled by CMV infection through periodic episodes of reactivation from its latent state. Memory T cell inflation appears to require T cell costimulation (68, 69), yet is less dependent on the immunoproteasome (70). Modifying the context of the peptide can convert a classical response to an inflationary one (71).

Recombinant CMVs may provide important vectors for vaccines, although they are highly complex viruses containing multiple immune evasion genes. Nevertheless, in experimental models engineered mouse cytomegalovirus (MCMV)-based vaccine vectors containing foreign viral sequences (e.g., derived from influenza virus, lymphocytic choriomeningitis virus, Ebola virus, herpes simplex virus, and respiratory syncytial virus) provide long-lasting protection (42, 71–73). In rhesus macaques, a recombinant CMV vector expressing simian immunodeficiency virus (SIV) antigens induced in addition to MHC class I-restricted CD8^+^ T cell responses also MHC class II-restricted and HLA-E-restricted CD8^+^ T cell responses (74, 75). These unconventional responses are likely to arise because of the restrictions placed on normal antigen presentation by the attenuated CMV vectors used. More work is needed to identify which of these populations is critical for protection, and whether this protection correlates to magnitude, breadth, or effector mechanism.

Memory inflation is not exclusively induced by CMV. Similar phenomena have been observed with other viruses, e.g., Epstein–Barr virus (EBV), herpes simplex virus-1, parvovirus B19, murine polyoma virus, and adenoviral vectors (66, 76). The latter is of interest with respect to vaccine-induced responses. In mouse models, adenovirus-based vectors can lead to induction of inflationary responses, which closely resemble those induced by natural CMV infections (77, 78). Moreover, in this vaccine platform, it is possible to generate inflationary responses against otherwise non-inflationary epitopes by constructing “minigenes,” in which only the CD8 T cell epitope of interest is inserted into the vector and expressed, thus bypassing antigen processing requirements (79). Adenoviral vectored vaccines have been developed against many pathogens, including EBV, HCV, HIV, malaria, and Ebola (4, 64, 80–82), and the responses elicited by these vectors in human volunteers are sustained over time. The HCV-specific responses induced in healthy CMV^+^ volunteers after immunization with a chimpanzee adenovectored-HCV vaccine shared similar phenotype and functionality to their CMV-specific memory populations as well as to inflating memory cells induced after AdHu5 and MCMV infection in mice (78).

## The Breadth of the Induced T Cell Response Impacts on Protection

An increased breadth of the vaccine-induced T cell response has been found beneficial against many chronic viral pathogens (5, 54, 83–86). Induction of T cells with multiple antigen-specificities correlates with advanced killing capacity for control of HCV or even complete eradication during primary infection with HCV and superior protection upon reinfection (80, 86, 87). Analysis of CD8^+^ T cell responses in untreated HIV-infected individuals showed that an increasing breadth of Gag-specific responses is associated with decreased viremia (88).

Successful induction of potent and broad T cell responses has been reported for DNA plasmid vaccines (89, 90) and adenovirus serotype 26 vector-based vaccines (91). The latter approach incorporated a combination of subdominant and dominant epitopes of rhesus macaques SIV in prime-boost vaccination schedules. In parallel with these findings, the efficacy of synthetic long peptide (SLP)-based vaccines to protect against MCMV was significantly improved by combinations of SLPs that increased the breadth of the antigen-specific T cell response (5). These findings indicate that cytotoxic CD8^+^ T cell populations consisting of a broad repertoire of specificities are better capable to effectively kill virus-infected cells compared with T cell pools with a single specificity. Possible explanations are that T cells of diverse specificity results in enhanced killing of virus-infected cells (compared with T cells with one specificity) or that viral escape mechanisms become restricted. Moreover, an increase in recognition of multiple epitopes may also contribute to protection against infection with heterologous viruses *via* cross-reactive responses (92). Vaccine efficacy is expected to be also dictated by the TCR clonotypes within a polyclonal antigen-specific T cell population, since immune escape during viral infection is linked to conserved TCR motifs while diverse clonotypic repertoires without discernible motifs are not associated with viral escape (93, 94). Hence, the importance of the diversity in the antigen-specific T cell repertoire (with respect to recognition of multiple antigens and diversity in clonotypes specific for the same epitope) should be taken into account while designing prophylactic T cell-based vaccines.

As discussed earlier, both the magnitude and breadth of the T cell response is of importance. However, it should be noted that simply determining the magnitude in the blood is not always valuable, as vaccine efficacy depends also on the type of memory T cell and its location. For example, a direct association between protection and the frequency of the T cells in the circulation does not always exist (95). Actually, depending on the route of infection, T cells present in the mucosal surfaces or in the tissues (T_EM_ and/or T_RM_) play a dominant role in controlling the infection, and sufficient numbers in these areas rather than in the circulation are likely required to form a robust frontline defense against, e.g., HIV-1 (30, 96). Competition between antigens (e.g., the cellular processing and presentation machinery) is also an important consideration (5), highlighting that antigen selection is not simply a case of “the more the better.” Furthermore, not all antigen-specific T cell populations have the same efficacy on a per-cell basis. For example, T cell populations specific for CMV antigens that invoke inflationary responses show superior protective capacity (5). Selection of the correct but also the appropriate quantity of antigens will ultimately steer the immune response and is thus a very critical step of the vaccine development process. Especially, antigens provoking antigen-specific T cell populations with enhanced magnitude, breadth, and diversity in the clonotypic repertoire should be tested and subsequently selected for inclusion when designing vaccine vectors or synthetic vaccines. Furthermore, there is evidence that, besides the quantity and breadth, specific features of antigen-specific T cell populations such as their cytokine polyfunctionality and metabolic properties are also of crucial importance for vaccine efficacy, and this will be further discussed in the next sections.

## Cytokine Polyfunctionality of T Cells as Parameter of Vaccine Efficacy

Cytokine production is an important effector mechanism of T cell-mediated immunity. Upon most viral and bacterial infections protective T cell immunity consists of CD4^+^ and CD8^+^ T cells with a “Th1” cytokine profile that is characterized by (co-)production of IFN-γ, TNF, and IL-2 (97).

The frequency of IFN-γ-producing T cells has been widely used as a parameter to assess vaccine-induced responses. In terms of effector function, IFN-γ has been shown to play a role in the clearance of various viral infections (98). However, there are many examples showing that the magnitude of the IFN-γ secreting T cell response is not a sufficient immune correlate of protection. Single positive IFN-γ-producing T cells can comprise a relatively large fraction of the total cytokine-producing CD4^+^ and CD8^+^ T cell population after immunization. However, such T cells have a limited capacity to be sustained as memory T cells (99). Hence, prophylactic vaccines that elicit a high proportion of single IFN-γ-producing T cells would likely not be protective and provide a clear example for why the quality of the response is far more useful in assessing long-term protection than just measuring the frequency of IFN-γ-producing T cells. Instead, studies characterizing (vaccine-elicited) T cell responses against HIV, HBV, HCV, CMV, influenza, and *Leishmania* revealed a strong correlation between the protection level and the induction of high frequencies of polyfunctional T cells [e.g., coproducing IFN-γ, TNF, and IL-2 (4, 80, 100–107)]. Importantly, some of these studies showed that measuring the magnitude of IFN-γ-producing CD4^+^ and CD8^+^ T cells alone was not sufficient to predict protection, and provided evidence that measuring the quality of the CD4^+^ and CD8^+^ T cell response, *vis-à-vis* polyfunctional T cells, is required.

The supremacy of the polyfunctional T cells may relate to the superior survival properties of these cells (81, 99, 108) and to a higher level of target killing (109). This may be related to a higher IFN-γ production on a per-cell basis by polyfunctional cells compared with monofunctional cells (110), and to the capacity of TNF that is like IFN-γ also capable of mediating the killing of virus-infected cells (111–113). Moreover, reciprocal production of IFN-γ and TNF leads to synergistic actions (114).

**Figure 1:**
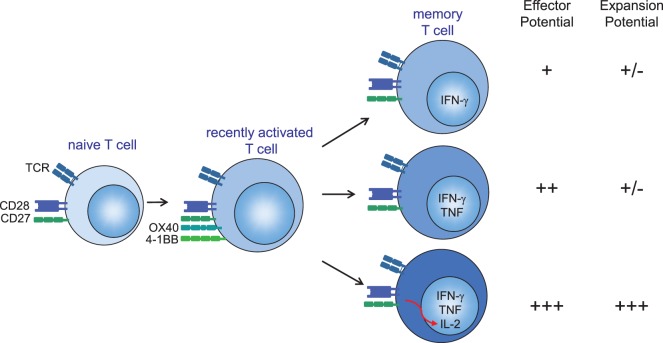
Several mechanisms account for the optimal protection of (vaccine-elicited) polyfunctional cytokine-producing CD4^+^ and CD8^+^ T cells. (1) Secretion of more IFN-γ on a per-cell basis. (2) T cells secreting both IFN-γ and TNF have enhanced effector activity compared with T cells that secrete IFN-γ alone. (3) Autocrine interleukin (IL)-2 production promotes the secondary expansion of memory T cells. Hence, IL-2, TNF, and IFN-γ provide a relatively simple set of cytokines that can be used to define a vaccine-elicited response against specific infections that require T cells for protection. CD28 signals are required for sufficient T cell priming during the primary phase of an infection, while OX40 (CD134) and 4-1BB (CD137) gain importance during the late effector and memory stages of antigen-specific T cells either by providing pro-survival signals or by enhancing the quality of the memory T cells (127, 128). CD27 stimulation is important during both early and late phases after infection (68). T cell costimulation *via* CD28 and tumor necrosis factor receptor family members (CD27, OX40, and 4-1BB) can provide signals to enhance autocrine IL-2 production.

Furthermore, the other cytokine in the panel, IL-2, is decisive as well. Studies analyzing the production of IL-2 and IFN-γ by CD4^+^ and CD8^+^ T cells from individuals infected with HIV showed that long-term non-progressors, or individuals on anti-retroviral treatment, had increased frequencies of T cells expressing IL-2 only or both IL-2 and IFN-γ, whereas individuals with high viral loads (progressors) have increased frequencies of T cells producing IFN-γ only (95). Although IL-2 has no direct antiviral function, it promotes proliferation and secondary expansion of antigen-specific T cells (115–120). In addition, IL-2 increases expression of the effector molecules perforin and granzyme, which mediate cytolytic function (121, 122). IL-2 signals may also enhance NK cell activity that could contribute to the early control of infection following challenge (99, 123–126). Taken together, we conclude that cytokine polyfunctionality is of major importance for the efficacy of T cell-based vaccines (Figure 1), hence dissecting how cytokine polyfunctionality is regulated during the programming of T cells is of interest and may reveal potential strategies to improve vaccine-mounted T cell responses.

## Improving Vaccination by Targeting T Cell Metabolism?

The transition of naïve T cells to active effector cells and memory T cells involves dynamic and coordinated metabolic modifications (129). This reprogramming of the cellular metabolism is not a consequence of activation but is linked to the differentiation and activation processes and reflects the fuel and substrates necessary to support the differentiation stages of a T cell (130, 131). Both naïve T cells and memory T cells rely primarily on oxidative phosphorylation (OXPHOS) and fatty acid oxidation (FAO) for fuel. This reflects the low level yet persistent need for energy as such cells are generally long-lived. Effector T cells on the other hand have particularly high energetic and synthesis demands. These cells have enhanced glycolysis and employ the mitochondrial tricarboxylic acid cycle to support their demand for *de novo* proteins, lipids, and nucleic acids synthesis. It is becoming increasingly clear that metabolic reprogramming plays a critical role in T cell activation, differentiation, and function. The distinct metabolic demands of different T cell subsets make them exquisitely sensitive to pharmacologic inhibitors of metabolism (132). These different metabolic requirements of T cell subsets provide us with a promising therapeutic opportunity to selectively tailor (vaccine-induced) immune responses. Thus, targeting T cell metabolism affords the opportunity to additionally regulate vaccine-induced responses.

Metabolic reprogramming occurs simultaneously with T cell activation and is facilitated by mTOR (mammalian target of rapamycin) (133). mTOR activation promotes glycolysis, fatty acid synthesis, and mitochondrial biogenesis. As such, targets upstream and downstream of the mTOR signaling pathway are potential therapeutic targets. Rapamycin, although known as an “immunosuppressive” drug due to its ability to slow down T cell proliferation, promote robust responses to vaccination by enhancing CD8^+^ T cell memory formation (134). Correspondingly, deletion of the mTORC1 inhibitory protein TSC2 leads to enhanced mTORC1 activity and increased effector function (135). Targeting of TSC2 or other molecules in the mTOR pathway might accordingly enhance immunity.

Targeting of glycolysis to inhibit immune responses in the setting of autoimmune disease and transplantation rejection is evolving, and this strategy is also used to enhance antitumor immunity by promoting long-lived memory cells *ex vivo* (136). Whether this can be used in vaccination strategies remains to be examined. Although most studies have focused on the critical role of glycolysis in promoting effector T cell generation and function, it has become clear that mitochondrial-directed metabolism also plays an important role. Memory T cells rely for their energy upon OXPHOS and FAO. Because these metabolic pathways are dependent on mitochondria, the abundance and the organization of the mitochondria are instrumental for development of fit memory cells (137). Alterations in the mitochondrial biogenesis can influence the differentiation of T cells, thereby providing opportunity to augment T cell-mediated immunity (138, 139). The transcription factor PGC1α promotes mitochondrial biogenesis and function (140). Hence, pharmacologically or genetically enhancing PGC1α represents a potential strategy for improving vaccine-induced T cell responses. In *ex vivo* systems, it has already been shown that enforced overexpression of PGC1α, leads to improved metabolic fitness and effector cytokine function of CD8^+^ T cells (141). Finally, the immediate uptake of amino acids such as glutamine and leucine is critical for proper metabolic reprogramming of T cells. This is accompanied with the upregulation of amino acid transporters involved in glutamine (SLC1A5) and leucine (SLC7A5/SLC3A2 heterodimer) (142, 143). Whether *in vivo* targeting of the above described metabolic processes is possible remains to be examined and may depend on the specificity of metabolic inhibitors/enhancers as they could affect many cells of the body. The future will tell if indeed metabolic targeting is possible to enhance vaccines. Nevertheless, the metabolic profiles of (vaccine-induced) T cells are surely of interest and correlate to vaccine-mediated immunity (144).

## Costimulation Empowers T Cell-Eliciting Vaccines

Targeting costimulatory and inhibitory receptors on the cell surface of T cells has shown efficacy in various preventive and therapeutic preclinical vaccination settings. Costimulatory signals transduced *via* the CD28 family members CD28 and ICOS and *via* the tumor necrosis factor receptor (TNFR) family members CD27, 4-1BB, and OX40 play dominant roles in orchestrating the required “signal 2” for optimal T cell proliferation and survival (127). While CD28 and CD27 are constitutively expressed on naïve T cells, ICOS, 4-1BB, and OX40 are upregulated upon T cell activation (127, 145). Collaboration between costimulatory molecules was expected (127, 146) and confirmed in experimental models (147).

Enforced engagement of costimulatory molecules results in enhanced T cell activation, expansion, survival, and establishment of long-term memory (148–154), and has thus the potential to serve as effective immunomodulatory components of prophylactic vaccines against chronic viruses (127, 151, 155). Indeed, this has already been observed for DNA and adenovirus-based vector vaccines in which enforced expression of costimulatory ligands stimulating CD27, 4-1BB, and OX40 leads to increased T cell expansion, enhanced cytotoxic activity and antibody responses (156, 157). Strikingly, agonistic antibodies to OX40 combined with synthetic peptide vaccines prompt robust effector and memory CD4^+^ and CD8^+^ antiviral T cell responses, thereby enhancing the prophylactic vaccine efficacy against lytic MCMV infection (153). Chronic viral infections are characterized by accumulation of functionally impaired antigen-specific CD8^+^ T cells. Studies have shown that activation *via* 4-1BBL alone or in combination with CD80 can enhance the generation of primary CD8^+^ T cell responses and induce expansion of the antigen-specific CD8^+^ T cells from this pool of impaired T cells (145, 158). Similarly, 4-1BB stimulation has been shown to enhance the generation of primary CD8^+^ T cell responses (148, 159, 160) and synergizes with attenuated vaccinia virus vectors to augment CD8^+^ T cell responses (148).

Targeting of inhibitory molecules on T cells, such as PD-1 and CTLA-4, has been shown to restore the effector function of (over)activated T cells in settings of chronic viral infections and cancer (161–164). Inhibitor blockade with monoclonal antibodies in combination with therapeutic vaccines synergizes in reinvigorating antitumor and antiviral T cell responses (165, 166). Targeting of inhibitory pathways during primary immunization with prophylactic vaccines may advance the vaccine efficacy as well (167, 168).

Although the use of antibodies targeting costimulatory and inhibitory molecules as immunostimulatory modalities in vaccine approaches can facilitate antigen-specific T cell responses, the use of such Abs, however, is associated with toxicity as demonstrated in rodents and in clinical settings (164, 169–171). Nevertheless, given the potential benefit to significantly increase the effectiveness of vaccines, both the efficacy and safety of targeting costimulation is currently extensively examined in various immunotherapeutic approaches against persistent viral infections. Examining the timing and/or the dosing is in this respect an important aspect, not only to prevent unwanted side effects but also to improve effectiveness. However, mass deployment of antibodies to improve vaccines may be too expensive, hence alternative methods able to target costimulatory and inhibitory molecules are desired.

CD28-mediated costimulation modulates T cell metabolism *via* activation of PI3K pathways, and this is essential to control effector cytokine production (172, 173). Moreover, CD28 signaling leads to PI3K-dependent upregulation of surface GLUT1 to facilitate enhanced glucose influx (172). This upregulation of GLUT1 is critical for T cell function, as genetic deletion of GLUT1 markedly inhibits effector T cells (174). Concomitant with increased expression of glucose transporters is the upregulation of key glycolytic enzymes (175). The inhibitory receptor PD-1 also regulates metabolic activity including glycolytic and mitochondrial processes (139, 176). TNFR family members are also able to metabolically program T cells (177, 178). Another important property of T cell costimulation is its effect on improving the T cell cytokine polyfunctionality. For example CD28 but also the TNFR family members are able to promote IL-2 production (153, 179–181), thereby directly improving the cytokine polyfunctionality (Figure 1). The TCR affinity also impacts polyfunctionality (182), and likely the collective signals of the TCR and costimulatory receptors are programming the polyfunctional status of T cells. In conclusion, targeting of T cell costimulation can impact the important quantitative (magnitude, breadth) and qualitative (cytokine polyfunctionality and metabolic fitness) determinants of vaccine-induced T cells, and provides thus major opportunities for further exploration in future vaccine designs.

## Conclusion and Perspectives for Vaccine Design

The design of vaccines that imprint T cells with the ability to protect against persistent viral pathogens has gained remarkable progress. An understanding of the appropriate initial programming signals is a key step, as is how the route of priming or boosting influences the development of effective memory T cells. A combination of several metrics such as the type of the memory T cell, breadth, polyfunctional quality, and metabolic characteristics demonstrate a valid toolbox to define when a vaccine-elicited T cell response is protective. Information about the anatomic location, activation, and differentiation of memory T cells in lymphoid compared with non-lymphoid organs could be very valuable as well. Costimulatory signaling pathways mediate important T cell memory properties (e.g., programming of cytokine polyfunctionality and metabolism) and may serve as interesting targets for vaccine improvement. Insight into these pathways may identify the requisite pathways and potentially other targets to improve T cell-based immunotherapy. Coupling this to the identification of the best correlates of protection for persistent viral pathogens will foster the development of more effective vaccination regimes.

## Author Contributions

All authors contributed to the writing of this review.

## Conflict of Interest Statement

The authors declare that the research was conducted in the absence of any commercial or financial relationships that could be construed as a potential conflict of interest.
